# A flexible Python-based touchscreen chamber for operant conditioning reveals improved visual perception of cardinal orientations in mice

**DOI:** 10.3389/fncel.2022.866109

**Published:** 2022-10-10

**Authors:** Christopher Wiesbrock, Simon Musall, Björn M. Kampa

**Affiliations:** ^1^Systems Neurophysiology, Institute for Zoology, RWTH Aachen University, Aachen, Germany; ^2^Research Training Group 2416 MultiSenses—MultiScales, RWTH Aachen University, Aachen, Germany; ^3^Bioelectronics, Institute of Biological Information Processing-3, Forschungszentrum Jülich, Jülich, Germany; ^4^JARA BRAIN, Institute for Neuroscience and Medicine, Forschungszentrum Jülich, Jülich, Germany

**Keywords:** natural scenes, touchscreen chamber, behavior, operant conditioning, Python, orientation discrimination

## Abstract

Natural scenes are composed of a wide range of edge angles and spatial frequencies, with a strong overrepresentation of vertical and horizontal edges. Correspondingly, many mammalian species are much better at discriminating these cardinal orientations compared to obliques. A potential reason for this increased performance could be an increased number of neurons in the visual cortex that are tuned to cardinal orientations, which is likely to be an adaptation to the natural scene statistics. Such biased angular tuning has recently been shown in the mouse primary visual cortex. However, it is still unknown if mice also show a perceptual dominance of cardinal orientations. Here, we describe the design of a novel custom-built touchscreen chamber that allows testing natural scene perception and orientation discrimination performance by applying different task designs. Using this chamber, we applied an iterative convergence towards orientation discrimination thresholds for cardinal or oblique orientations in different cohorts of mice. Surprisingly, the expert discrimination performance was similar for both groups but showed large inter-individual differences in performance and training time. To study the discrimination of cardinal and oblique stimuli in the same mice, we, therefore, applied, a different training regime where mice learned to discriminate cardinal and oblique gratings in parallel. Parallel training revealed a higher task performance for cardinal orientations in an early phase of the training. The performance for both orientations became similar after prolonged training, suggesting that learning permits equally high perceptual tuning towards oblique stimuli. In summary, our custom-built touchscreen chamber offers a flexible tool to test natural visual perception in rodents and revealed a training-induced increase in the perception of oblique gratings. The touchscreen chamber is entirely open-source, easy to build, and freely available to the scientific community to conduct visual or multimodal behavioral studies. It is also based on the FAIR principles for data management and sharing and could therefore serve as a catalyst for testing the perception of complex and natural visual stimuli across behavioral labs.

## Introduction

Mice have become a major model organism in visual neuroscience. The accessibility of high-density electrophysiology and functional imaging techniques combined with a large genetic toolbox for measuring and manipulation of neural activity allows the study of neural circuit function in unprecedented detail. However, to study the neuronal basis of visual scene processing it is essential to explore how mice perceive their visual inputs. Natural scenes are composed of an inhomogeneous distribution of edge orientations with an overrepresentation of cardinal over oblique edges (Girshick et al., 2011). Correspondingly, it has been shown in humans and numerous animal models that orientation discrimination is also more precise around cardinal orientations, suggesting a stronger neural representation of these visual features (Appelle, 1972; Girshick et al., 2011). Indeed, two-photon imaging of neuronal responses in mouse primary visual cortex (V1) revealed larger numbers of responding neurons and stronger neural responses to cardinal gratings compared to obliques (Roth et al., 2012). However, it is unknown if mice also perceive cardinal orientations more strongly than humans (Girshick et al., 2011).

To answer this question, we, therefore, aimed to test if orientation perception in mice also reflects the statistics of natural scenes. A number of studies have tested visual perception in mice using the visual water-maze task (Brandeis et al., 1989; Prusky et al., 2000; Wang et al., 2016) or head-fixed in front of a computer screen or spherical dome (Dombeck et al., 2007; Andermann et al., 2010; Busse, 2018). However, both strategies impose stress on the animals and are labor-intensive and therefore difficult to perform in larger cohorts. Touchscreen-based operant chambers are a powerful alternative to study visual perception in mice, allowing behavioral testing with minimal animal handling and without involving additional stressors, such as head-fixation. They are also well-suited to study rodent models of psychiatric and neurodegenerative diseases (Horner et al., 2013) and have been shown to be more accurate in detecting early prefrontal dysfunction in mice compared to standard water-maze tasks (Van den Broeck et al., 2021). While head-fixed mice can also learn visual discrimination by using an active engagement task based in a virtual environment (Poort et al., 2015), using a touchscreen has the additional advantage that mice can directly and intuitively respond by touching the visual target. This allows mice to quickly learn visual tasks, even if complex combinations of visual stimuli are used to test their perception of natural scenes or global motion (Horner et al., 2013; Stirman et al., 2016; Yu et al., 2018).

However, commercial touchscreen chambers only allow a low number of task designs and restrict the use of complex visual stimuli. A recent approach, using a custom-built touchscreen chamber, used more complex visual stimuli, such as natural images, to reveal that mice are adept at discriminating natural scenes (Yu et al., 2018). Inspired by this work, we designed a touchscreen chamber for operant conditioning, based on the open-source Python framework. This framework allowed us to apply different strategies to test the visual discrimination capabilities of mice, including a staircase approach to determine the visual orientation discrimination threshold in fine detail, and a new parallel visual discrimination task allowing for within subject comparisons of different visual discrimination targets. Using our touchscreen chamber we revealed a cardinal orientation preference in mice, most likely reflecting the orientation distribution in natural scenes (Girshick et al., 2011) as well as the overrepresentation of cardinally tuned neurons in V1 (Roth et al., 2012).

To summarize our scientific motivation, we want to emphasize the usability of the proposed open-source touchscreen chamber and demonstrate its capabilities by flexibly addressing an important scientific question. Openly available methods can be used to flexibly tackle questions about sensory perception and behavior while increasing the reproducibility of behavioral results across different labs. With flexible and transferable compositions of hardware and software, we want to open a door for the whole community and reduce restrictions due to the high cost of commercial solutions. The proposed touchscreen chamber can therefore be an important part to answer questions in different frameworks, such as the sensory perception of naturalistic stimuli or multisensory integration.

## Materials and Methods

### Animals

All experiments were authorized by the local authorities (84-02.04.2016.A357, LANUV NRW). A total of 37 mice were used for this study and successfully trained in the touchscreen chamber. The pretraining and visual discrimination tasks were performed in three male C57BL/6 mice starting at the age of 8 weeks. Eight mice of the same strain and age, were trained in the parallel visual discrimination task (four males and four females) and 14 mice were trained in the staircase orientation discrimination task (learning conditions: six mice for cardinal, six mice for retraining, and four mice for oblique). To demonstrate the effect of the bias correction on reducing intrinsic biases and behavioral strategies, behavioral data from 12 adult, male animals during an orientation discrimination task was included. Here, six mice were trained with active bias correction and six mice without active bias correction. All mice were bred and kept at the animal facility of the institute. During the experiment, the animals were housed on a reversed Day/Night-cycle of 12 h:12 h. The animals were water restricted throughout the experiments. They received water *ad libitum* on 1 day of the week, on which no experiments were performed and received at least 1.5 ml of water on experimental days. Mice had access to food *ad libitum* and were weighed and checked for their health status before the start of each behavioral session.

### Setup

The design of the Touchscreen Chamber has been shared online^1^ including a detailed parts list. The setup consisted of polyvinyl chloride (PVC) plates in a trapezoidal shape as described in previous studies (Bussey et al., 2008; Mar et al., 2013). Within the screen holder, a display of 11 inches (ELO touch 1002L) with an infrared touchscreen frame (NJY touch, Guangdong, China) was mounted in the front. The infrared frame sent the input information, i.e., mouse touches, *via* the USB port to the computer system. The infrared frame is crucial, since the touches of the mouse were not accurately detected by a capacitive or resistive touchscreen. On the opposite side of the screen holder, the wall contained the water delivery spout and a green LED light indicating correct responses to the animal. To provide acoustic feedback to the animal two loudspeakers were placed next to the screen outside of the animals’ arena.

### Visual stimulation

**Figure 1:**
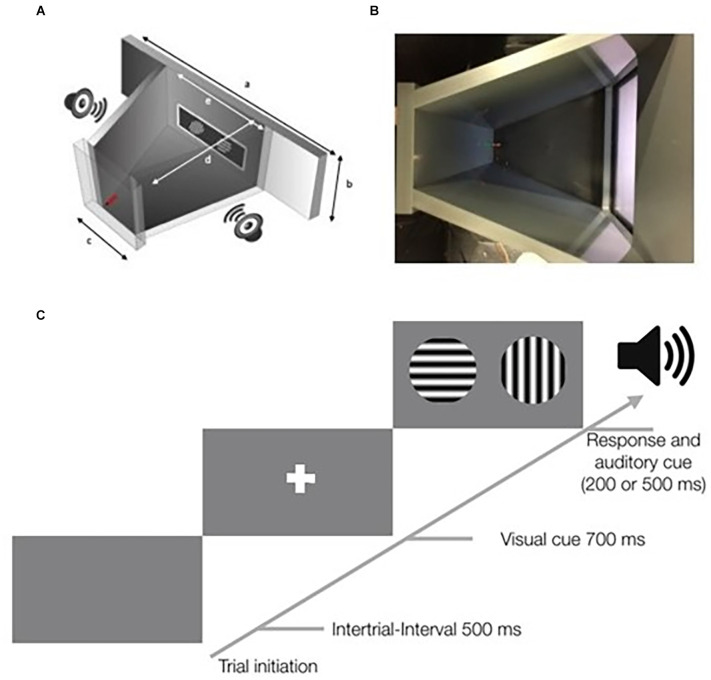
Schematic overview of the touchscreen chamber and a schematic of the stimulus protocol. **(A)** Overview of the touchscreen chamber. A screen with a touchscreen frame was placed in a drilled hole. The screenholder has dimensions of 410 mm × 273 mm (a). The dimension within the training area of the screenholder is 165 mm × 273 mm (e). Outside of the closed training area (gray) two loudspeakers were placed on each side. On the opposite wall [47 mm × 273 mm (c)] to the screen, a red water spout was placed below a green LED light. The distance between is 160 mm (d). Further information on the dimensions of the setup, including a 3D-model, is available in the online repository. **(B)** Top view of the touchscreen chamber. On the right side, the touchscreen is shown. On the left side, the green LED and the red water spout are visible. The viewing angle is similar to that of a webcam that was used to record the mouse in each session. **(C)** Scheme of a typical trial in the touchscreen chamber. Mice initiate trials by touching the water spout. After a 500-ms delay, a visual cue in form of a white cross is shown for 700 ms. Subsequently, the target and the distractor stimuli are displayed. After the animal responds by touching one of the two stimuli, an auditory cue occurs. A correct response is followed by a short low-pitched tone of 200 ms, while an incorrect response is followed by a longer high-pitched tone of 500 ms.

Visual stimuli were presented *via* a computer with an installation of PsychoPy v1.83.04 (Peirce, 2007, 2009). The display, touchscreen frame, data acquisition device (1208LS, Measurements Computing), webcam. and loudspeakers were connected to the same computer. All touch signals were registered and correct responses caused a trigger to be sent to the data acquisition device which switched on the green LED and played a low-pitched tone on the loudspeakers. After false responses, a bright white screen and a high-pitched tone were presented. Correct stimuli (target, S+) and incorrect stimuli (distractor, S−) were predefined and can be varied between projects. Before the stimuli were shown, a white cross as a visual cue is presented for 700 ms (Figure 1C). We used sine-wave gratings of different orientations. The stimuli had a diameter of 7 cm during all experiments. The spatial frequency was set to 0.04 cycles/degree measured from the center of the touchscreen chamber. The screen resolution was 640 × 480 pixels with a Michelson contrast of 0.98 for the used screen.

A piezo element on the waterspout was used to register animal licks. The amplified signal from the piezo element was recorded with the data acquisition box and triggered licking signals after passing a preset threshold. After a correct response, the lick detection triggered the magnetic valves to release a water reward of 0.025 ml. After a false response, the animal had to lick the waterspout again to deactivate the white light punishment and initiate the next trial. The webcam recording was started together with the experiment to record the behavior of the mouse during the full experimental session.

### Behavior control

All Python software for stimulus presentation and setup control are provided in a public Git-repository^1^. When the animal touched the screen, the software compared the touch location with the location of the reward stimulus. In case of a correct response, the software sent a trigger to induce the onset of the green LED and activated the lick detection. If the stimulus presentation was active for 2 min and the infrared touchscreen did not register any responses, the experiment was terminated due to the inactivity of the mouse.

### Pretraining

**Figure 2:**
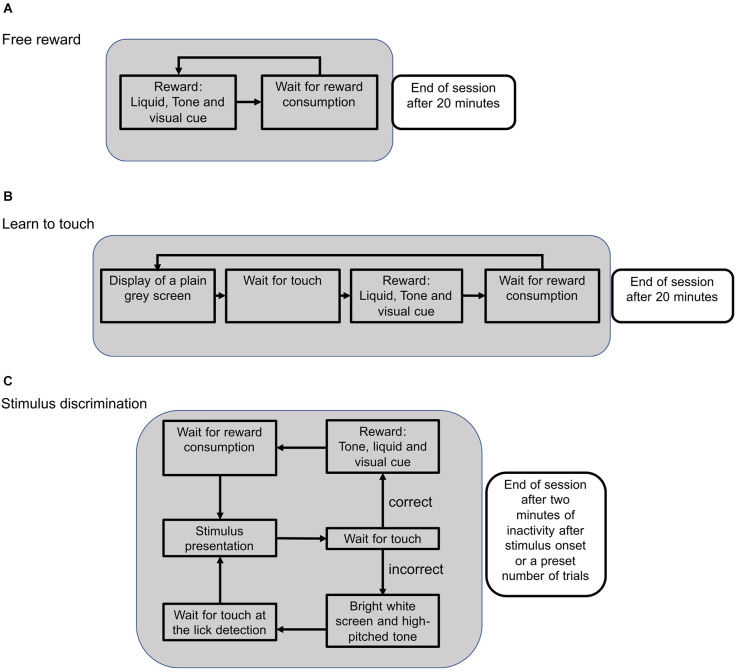
Schematic flow of an experimental session during different training phases. **(A)** During the reward training, available rewards were indicated by a green LED as a visual cue. When the lick detection was triggered, the green LED turned off and the trial was restarted. Sessions were stopped after 20 min. **(B)** In the subsequent training phase, the mouse had to touch the screen in order to activate the reward system. After touching the screen, the green LED turned on and a reward could be collected to initialize the next trial. **(C)** During the stimulus presentation, two different stimuli were presented and the animal could either touch the target or the distractor stimulus. Touching the target position caused the onset of a visual cue and a low-pitched tone. With the consumption of the reward, the next trial was initialized. Touching the distractor position was followed by a high-pitched tone and a bright screen to signal an error. The next trial needed to be initialized by triggering the lick detection without getting a reward.

After mice were habituated to the touchscreen chamber for 30 min on the first day, the pretraining consisted of three different phases (Figure 2). First, mice collected free rewards to associate a visual cue from the green LED with a water reward. Water was only available when the LED was switched on (Figure 2A). After mice collected at least one reward per minute on two consecutive sessions, the next training phase was initiated. Here, mice had to touch the screen in order to trigger the green LED and collect a water reward (Figure 2B). After the animals reliably touched the screen to trigger rewards (at least once per minute), they were trained on a visual target, such as a horizontal target next to a vertical distractor stimulus.

### Visual discrimination task

Two visual stimuli were presented simultaneously to train the mice to distinguish between different grating orientations (Figure 2C). According to the learning conditions, we used different stimulus sets. For the cardinal target condition, this was a horizontal target (S+) next to a vertical distractor (S−) stimulus. The session was ended if the animal stopped responding for more than 2 min or reached a maximum of 100 trials. The position of the target and distractor stimuli was randomly assigned to be on the left or right side of the screen. If the animal exhibited a behavioral bias, such as repeating responses on the same side, the stimulus presentation was adjusted to counteract the bias (see also “Response bias correction” Section below). In the oblique target condition, we showed two obliquely orientated sine-wave gratings with a 90° orientation difference at 45° (S+) and 135° (S−).

### Retraining

To test, whether animals can adapt to new task conditions, we introduced a retraining paradigm. During the retraining, we used animals which had already learned the cardinal target condition and retrained them with oblique stimulus sets. During this retraining phase, we presented the distractor stimulus with orientation differences of 22.5° and 45°. Even though the animals reached expert levels of 80% performance or higher in their initial training with cardinal stimuli, we had to lower the expert threshold during the retraining paradigm to 70% because mice were unable to achieve the same performance levels during retraining.

### Staircase visual discrimination task

The animals performing in the staircase visual discrimination task, were pretrained as described above (Figure 2) and then trained to reach expert performance in the visual discrimination before the introduction of the the staircase procedure. In the staircase visual discrimination task, a target was shown next to a dynamically oriented distractor stimulus. The orientation difference between target and distractor was based on the animals’ performance, using a 3-up/8-down rule: every correct response decreased the orientation difference by 3°, whereas every incorrect response increased the orientation difference by 8°. This adaptive procedure leads to a convergence of the orientation difference between target and distractor around the orientation discrimination threshold of the animal. To determine the orientation threshold in each session, we calculated the average orientation difference at the turning points when the rule was flipped from decreasing to increasing the orientation difference or *vice versa*. Figure 5 shows two example sessions to visualize this procedure.


(1) Steps up ∗ p=Steps down ∗ (1−p)

(2) $$p=\frac{1}{\left(1+\frac{Steps\;Up}{Steps\;Down}\right)}$$

**Figure 3:**
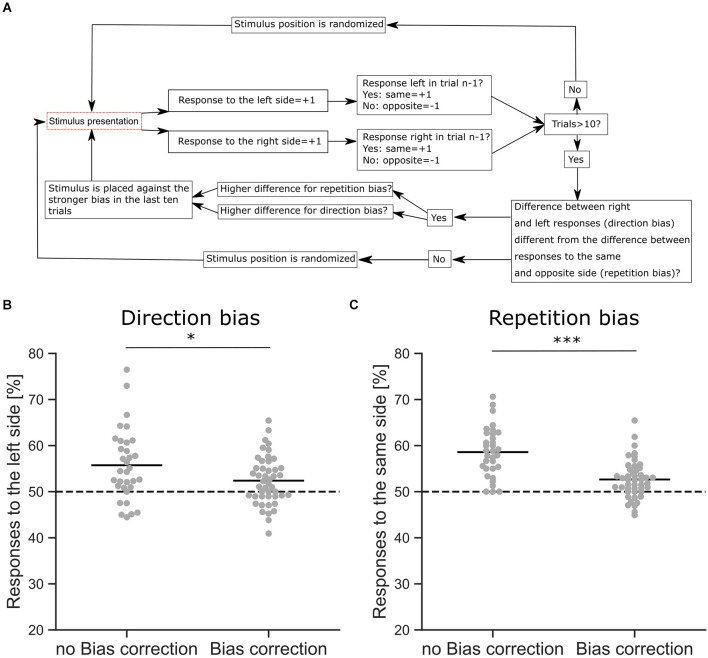
Response bias correction. **(A)** Schematic overview of the bias correction algorithm. **(B)** Comparison of a direction bias with (*n* = 46 sessions from six mice) and without bias correction (*n* = 33 sessions from six mice), shown as the proportion of responses to the left side. The difference between the proportions is significant (z-Test, *p* = 0.042). **(C)** Comparison of a repetition bias with (*n* = 46 sessions from six mice) and without bias correction (*n* = 33 sessions from six mice), shown as the proportion of responses to the same side as in the trial before. The difference between the proportions is significant (z-Test, *p* < 0.001). *p < 0.05, ***p < 0.001.

**Figure 4:**
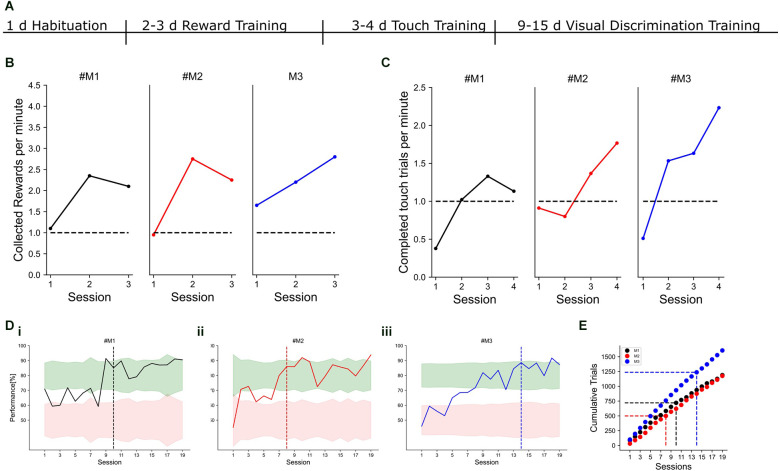
Example learning curves. Individual mice are color-coded in black, red, and blue. **(A)** Timeline of training phases and their respective durations. **(B)** Performance measured in collected rewards per minute for three example mice. The criterion to reach the next training phase was set to one collected reward per minute. **(C)** Performance during touch training. Here, the screen had to be touched to receive a water reward. The criterion to reach the next training phase was set to one completed trial per minute. **(Di–iii)** Visual discrimination performance with a horizontal target and a vertical distractor for three different mice. The target criterion for expert task performance was set to 80% correct responses for two consecutive days. The session, in which the criterion was reached is indicated by the dashed line. Red and green shading show 95% confidence intervals for chance and expert performance, respectively. **(E)** Cumulative trials for the three example mice. The dashed vertical line indicates the session, in which the expert criterion was reached. The horizontal dashed line indicates the number of trials, which were needed to reach the criterion.

**Figure 5:**
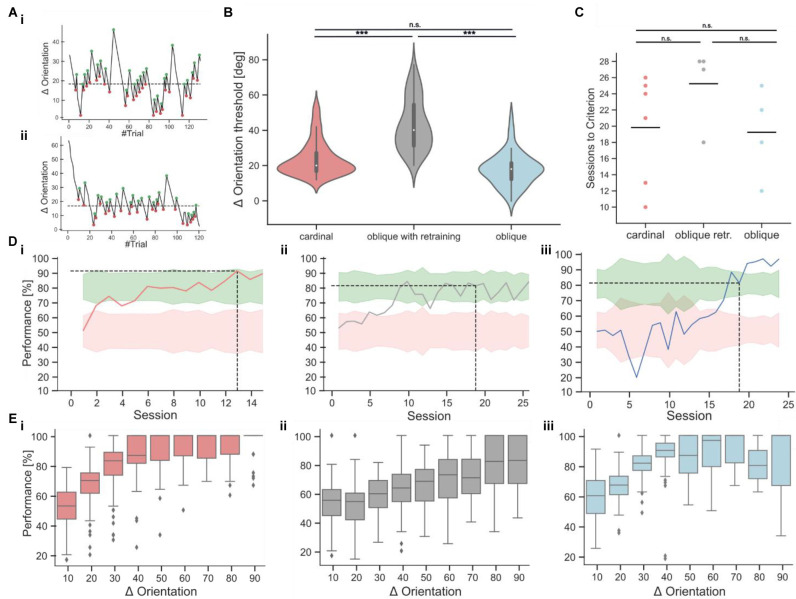
Orientation discrimination with cardinal or oblique target stimuli. **(Ai,Aii)** Two example sessions from the staircase experiment, showing fluctuations in orientation differences over the course of each session. The turning points, used for the calculation of the threshold are indicated as green and red dots. The dashed line indicates the threshold as the average of all turning points. **(B)** Orientation discrimination thresholds for cardinal targets (137 sessions from six mice), oblique targets after retraining (69 sessions from four mice), and oblique targets without retraining (52 sessions from four mice). Discrimination thresholds with a cardinal target were significantly lower compared to oblique targets after retraining the same animals (Kruskal-Wallis-Test, *p* < 0.001). Animals that were directly trained on oblique targets, had similar discrimination thresholds as animals trained on a cardinal target (Kruskal-Wallis-Test, *p* = 0.62) and lower discrimination thresholds as retrained animals (Kruskal-Wallis-Test, *p* < 0.001). **(C)** Comparison of the training duration in sessions until the mice reached expert performance. The differences were not significant (Kruskal-Wallis-Test, *p* = 0.15). **(Di–iii)** Example learning curves from animals in the different task conditions, following the color code from 5C. The dashed line indicates after how many sessions the expert threshold of 80% performance was reached on two consecutive sessions. **(Ei–iii)** Psychometric curves derived from the staircase experiment for the cardinal (red), retraining (gray), and oblique (blue) training condition. The bin size is 10° and the x-axis indicates the upper boundary of each bin. ***p < 0.001. No significant results are indicated by “n.s.” (*p* => 0.05).

Following a 3-up/8-down rule, the discrimination performance threshold is set to be 72.7% (p). Every time the mouse is responding correctly, the difficulty is increased by decreasing the orientation difference by 3° (Steps up). When the mouse is responding incorrectly, the difficulty is decreased by an increase of the orientation difference of 8° (Steps down). With the staircase visual discrimination task, the orientation discrimination threshold is therefore defined as the orientation difference, at which the animals reach this performance level. By calculating the average of the turning points, it is possible to read out at which orientation difference a performance of 72.7% is reached.

### Parallel visual discrimination task

To test for within-subject differences in orientation discrimination, we designed a parallel visual discrimination task in which cardinal or oblique stimulus sets were randomly distributed over all trials. The cardinal stimulus set consisted of a horizontal (S+) and a vertical (S−) grating while the oblique stimulus set had the same 90° orientation difference but orientated at 45° (S+) and 135° (S−). Before the discrimination training, the animals received the same pretraining as described above (Figures 2A,B) and we ensured that mice were never exposed to cardinal gratings alone to avoid a behavioral bias. To minimize the risk of any choice bias, the parallel visual discrimination learning started immediately after the animals learned to touch the screen.

### Response bias correction

Mice often develop different behavioral strategies to solve a given task, resulting in separate response biases. A common strategy is to select a preferred response side, leading to a direction bias where choices are repeatedly made towards the same side regardless of the stimulus. The opposite strategy is also found, where mice exhibit an alternating bias and change their response side from trial to trial. Mice also tend to repeat choices that were rewarded previously, leading to a choice repetition bias (Akrami et al., 2018) that is also commonly observed in humans (Urai et al., 2017; Talluri et al., 2018). Since these strategies are independent of the sensory stimulus and therefore interfere with discrimination performance, we applied an automated bias correction algorithm based on previous reports (Knutsen et al., 2006; Figure 3). In the first 10 trials, the stimulus presentation was randomized with target stimuli occurring on either the right or left side. After 10 trials the bias correction was activated, comparing the number of responses on the left and right sides and whether there was an alternating response pattern. If a response bias was found, the stimulus presentation was counterbalanced to reduce the bias, e.g., if the mouse had a tendency to respond more to the left side, the target stimulus was shown on the right side (Knutsen et al., 2006). In rare cases, where all responses occurred to one side, a repetition bias would be detected and corrected for. The goal of the algorithm was to detect and discourage response biases by presenting the target stimulus on the non-preferred side (Figure 3A). Our bias correction successfully reduced direction biases during the visual discrimination task (Figure 3B, z-Test, *p* = 0.042). In the absence of such bias correction, we observed strong response biases, whereas the active bias correction significantly reduced these behavioral biases (Figure 3C, z-Test, *p* < 0.001). However, although the bias correction successfully reduced response biases of the mice, a small degree of repetition (z-Test against Chance, *p* < 0.001) and direction bias (z-Test against Chance, *p* < 0.01) was still present in the behavioral data (Figures 3B,C).

### Statistical analysis

No data was excluded from the statistical analysis. The presence of behavioral biases was tested with a z-test of each proportion against 50% chance level. We tested for the difference between behavioral biases with and without an active bias correction with z-tests for two proportions in each bias condition (direction and repetition bias). For the analysis of learning curves, the 95% confidence interval from a binomial distribution was computed according to the number of trials around the chance level of 50% and for the expert level of 80%. The difference in orientation discrimination thresholds was tested with a Kruskal-Wallis test. Group-by-group comparisons were performed with Wilcoxon tests. To compare the different learning curves in the parallel visual discrimination learning task, we divided the sessions into early (first five sessions), middle (following 10 sessions), and late sessions (last four sessions). We compared the performance during the three defined stages with Wilcoxon tests. All statistical tests were Bonferroni-corrected if multiple comparisons were performed.

## Results

### A flexible Python-based touchscreen chamber for operant conditioning

To test the perception of complex visual stimuli in freely moving mice, we designed a custom-built touchscreen chamber inspired by a recent report on natural scene discrimination in mice (Yu et al., 2018). Our goal was to establish a common platform for a wide range of behavioral tests and complex visual stimulus presentations. Moreover, we focused on using open-source software and providing detailed descriptions of our setup design to facilitate the sharing and reproduction of our approach and behavioral results by other labs. Figure 1 shows our setup design with a trapezoid chamber to focus the animal’s attention towards the touchscreen. Responses are directly made by touching the chosen visual stimulus on the screen. However, water reward is given on the opposite side of the chamber, forcing the mouse to move away from the response location. This has the advantage that the mouse is always in the same position when it returns to the screen at the beginning of each trial.

Visual stimuli were presented using PsychoPy, a Python package which is widely used in psychophysics experiments and offers a large range of off-the-shelf tools for users with little programming experience (Subramanian et al., 2011; Siegle et al., 2021). PsychoPy allows presenting any visual stimulus or stimulus combination on the screen which we leveraged in other projects to present different static or drifting gratings as well as complex naturalistic visual stimuli (Balla et al., 2021). For this study, we focused on a 2-alternative forced choice task (2-AFC) but more complex choices, odd-one-out or attention tasks are equally possible. In particular, our custom-written flexible software allowed the introduction of custom task designs, such as *Staircase* or *Parallel Visual Discrimination*, which would not have been possible with current commercially available chambers. Screen touches and licks on the water spout were acquired using custom-written Python software and the animals’ behavior was additionally recorded with an overhead camera. The same software also controlled the experimental flow (Figure 2). All software tools and hardware designs are shared on an open repository, including a detailed description of the setup with example data that allow other users to reproduce the methods and experiments shown here (see Section “Methods”).

### Discrimination of cardinal and oblique orientations

Cardinal orientations are overrepresented in our environment and many animals, including humans, show a corresponding increased discrimination performance for cardinal orientations compared to obliques (Appelle, 1972; Girshick et al., 2011). Whether mice have a similar perceptual bias has not been tested so far, but neurons in mouse V1 also show preferred responses to cardinal orientations (Roth et al., 2012). To reveal if the overrepresentation of cardinally-tuned neurons in the mouse visual cortex is reflected in an enhanced perception of cardinal orientations, we trained mice to discriminate a visual grating with a fixed target orientation from a simultaneously presented distractor grating on the touchscreen (Figure 1). After 1 day of habituation to the touchscreen chamber, mice quickly learned to obtain water rewards by licking the water spouts and associated touching the screen with the reward (Figure 4). After 1 week of pretraining, mice started with the visual discrimination task with two simultaneously presented visual grating stimuli. In the first set of experiments, a horizontal grating was chosen as a target against a vertical distractor with a 90° orientation difference. Mice learned to touch the target stimulus and reached stable performance levels above 80% correct choices within 2 weeks (Figure 4D).

Subsequently, we introduced a staircase approach in which distractors with deviating angles between 3° and 90° were presented (Figure 5A) to measure the orientation discrimination threshold at which mice fail to correctly identify the rewarded horizontal target stimulus (average threshold: 23.82 degrees ± 0.84, *n* = 137 sessions from six mice; mean ± s.e.m.; Figure 5B). We then retrained mice on an oblique target grating at an orientation of 45°. Mice achieved stable performance levels above 70% after 10–15 sessions. However, discrimination with oblique targets required larger orientation differences between target and distractor gratings for accurate task performance, resulting in significantly higher orientation discrimination thresholds (average threshold: 43.07 degrees ± 1.74, *n* = 69 sessions from four mice, Figure 5B). To test if the higher orientation discrimination thresholds for oblique target gratings might be due to the initial training on cardinal target gratings, we performed the same experiments in a new batch of mice that were immediately trained on oblique targets. Here, we found no significant difference in orientation discrimination thresholds (average threshold: 23.73 degrees ± 1.16, *n* = 52 sessions from four mice) compared to the first batch of mice that trained and tested on cardinal targets (Figure 5B). The number of sessions which were required to learn the task was also not significantly different between both cohorts but showed a large variance across individual mice (Figures 5C,D). To further validate the orientation discrimination thresholds, we also derived psychometric discrimination curves from the staircase experiment (Figure 5E). As expected, the discrimination performance was high for large orientation differences and decreased for orientation differences below the calculated orientation discrimination thresholds. Note that due to the design of the experiment, most of the trials occurred around the average orientation discrimination threshold (Supplementary Figure 1A). We also tested if the response delay would be shorter for easier trials in the staircase experiments but found no clearrelation between response delays and task difficulty (Supplementary Figure 1B).

### Orientation discrimination with parallel cardinal and oblique stimulus sets

**Figure 6:**
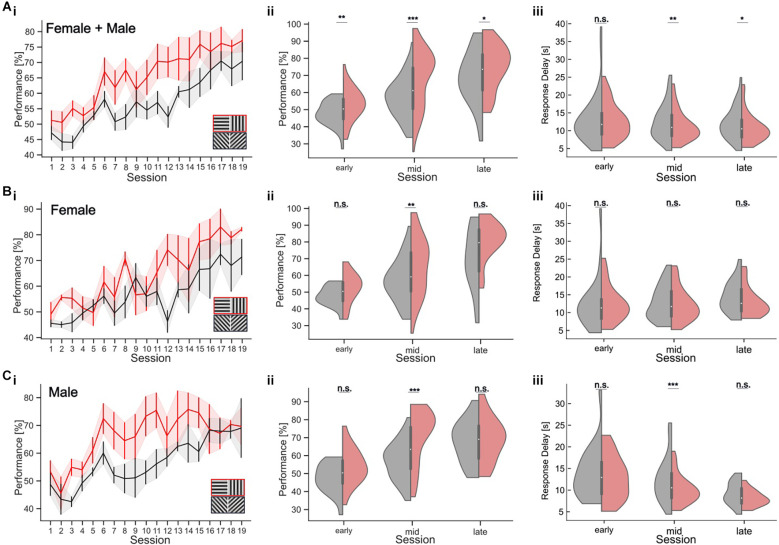
Orientation discrimination with a parallel visual discrimination task. Cardinal or oblique stimuli are shown in randomized order across trials. Trials with a cardinal target also had cardinal distractor, while oblique targets were shown with an oblique distractor at a constant orientation difference of 90°. **(A)** (i) Performance over sessions (*n* = 8 mice). The learning curves for the cardinal (red) and oblique stimuli (black) show the mean performance per session across all mice. Shaded areas indicate the SEM. The inset shows the two stimulus pairs. (ii) Performance in early, middle, and late periods of learning, for cardinal (red) and oblique (gray) stimuli for all mice. (iii) Response delay in early, middle, and late trials for cardinal (red) and oblique (gray) stimuli for all mice. **(Bi–iii)** Task performance and response delay for female mice only (*n* = 4 mice). Conventions as in **(A)**. **(Ci–iii)** Task performance and response delay for male mice only (*n* = 4 mice). Conventions as in **(A)**. *p < 0.05, **p < 0.01, ***p < 0.001. No significant results are indicated by “n.s.” (*p* => 0.05).

Our results show that training history has an impact on the animals’ orientation discrimination threshold, prohibiting a direct within-subject comparison after consecutive retraining from cardinal to oblique targets. In addition, comparing discrimination thresholds with oblique vs. cardinal orientations across different animals could be confounded by inter-individual variability in task performance and strategy. To directly test orientation discrimination with cardinal and oblique stimulus sets, we, therefore, established a new parallel visual discrimination task (Figures 6; see Section “Methods”). This is a variant of a paired association learning task (PAL) where mice learn to discriminate two pairs of stimuli: either two different cardinal or two different oblique gratings. This allowed us to test the discrimination performance with cardinal and oblique targets within the same subjects. To test the training effect for the different targets, we analyzed task performance during different periods of training (Figures 6Ai–iii). During the first five training sessions (“early”), mice showed an average performance of 52.76 ± 1.45% (average ± SEM) for the cardinal target and 47.30 ± 1.11% (average ± SEM) for the oblique target (Wilcoxon-Test, *p* = 0.003; Figure 6Aii). In the 10 subsequent sessions (“middle”), the task performance with cardinal targets was 67.93 ± 1.83% and 56.53 ± 1.31% with oblique targets (Wilcoxon-Test, *p* < 0.001). In the last five training sessions (“late”), mice reached their maximum performance with 75.1 ± 2.6% for cardinal targets and 68.7 ± 2.95% for obliques (Wilcoxon-Test, *p* = 0.02). To test whether the slower learning with oblique stimuli might be the result of a gender-specific difference in learning strategy, we separately analyzed the behavior of male and female mice (Jonasson, 2005). Male mice clearly learned the discrimination task faster with cardinal stimuli, which was visible as higher task performance during the middle sessions for cardinal targets (70.61 ± 2.33% in the middle sessions compared to 56.59 ± 1.67% for oblique targets; Wilcoxon-Test, *p* < 0.001; early: *p* = 0.051, late: *p* = 0.6; Figure 6C). The same effect was less pronounced but also significant for female mice (Figure 6B; Wilcoxon-Test, early: *p* = 0.11, middle: *p* = 0.006; late: *p* = 0.054).

Response delays also decreased over the course of training and differed between cardinal and oblique stimuli, especially in the middle of training. For all mice, the average response delay during the middle sessions was 11.09 ± 0.46 s for cardinal targets and 12.44 ± 0.55 s for oblique targets (Wilcoxon-Test, *p* = 0.004, early: *p* = 0.76, late: *p* = 0.029). This difference in response delay was only found in male mice, showing a response delay of 10.01 ± 0.51 s for cardinal targets and 12.36 ± 0.81 s for oblique targets during the middle sessions (Figure 6Ciii; Wilcoxon *p* < 0.001, early: *p* = 0.54, late: *p* = 0.1). The response delay of the female mice was not affected by the stimulus condition during the middle sessions, despite their higher performance with cardinal stimuli. The average response time was 12.16 ± 0.73 s for cardinal stimuli and 12.53 ± 0.74 s for oblique stimuli (Wilcoxon-Test, *p* = 0.47, early: *p* = 0.69, late: *p* = 0.11).

In summary, our results from the parallel visual discrimination task show that orientation discrimination performance in male and female mice is enhanced for cardinal vs. oblique orientations especially in the middle of training when performance increases were the largest. This is in agreement with earlier findings in humans (Appelle, 1972; Heeley et al., 1997; Furmanski and Engel, 2000; Girshick et al., 2011). However, prolonged training improved discrimination performance with oblique stimuli, leading to equal performance levels for both orientations in expert mice.

## Discussion

### Improved discrimination might reflect over representation of cardinal edges in natural scenes

We demonstrate the utility of our touchscreen chamber by testing mice in a series of visual orientation discrimination tasks and revealing an innate oblique effect where discrimination of cardinal gratings was enhanced compared to oblique orientations. This relates well to comparable results in other animal species (Appelle, 1972). Humans also show an increased discrimination sensitivity for cardinal orientated gratings, which has been linked to functional magnetic resonance imaging data that showed stronger V1 responses to cardinal vs. oblique orientations (Furmanski and Engel, 2000). Likewise, fitting cortical models to the visual discrimination behavior in humans proposed neuronal tuning properties in the visual cortex to fit with the natural statistics, in this case, the overrepresentation of cardinal edges (Girshick et al., 2011). In agreement with this hypothesis, 2-photon imaging studies in mice found an over-representation of cardinal-tuned neurons in V1 and to an even larger extent in the higher visual cortex (Kreile et al., 2011; Roth et al., 2012). Our finding of the oblique effect during the learning period in mice suggests that these neural tuning properties translate to enhanced perception of cardinal orientations, emphasizing that the mouse model is well suited to study the neural mechanisms of natural scene processing.

### Orientation discrimination and comparison of threshold and performance

The behavioral performance of mice in our touchscreen chamber was comparable to earlier reports. A previous study used touchscreen chambers to train mice on the discrimination of natural scenes and showed a similar number of sessions required for the animals to associate touching the screen with a reward (Yu et al., 2018). Mice also reached expert performance in 5–15 sessions, which is similar to our training results. A study that used a Go-/No-Go task with head-fixed mice found that mice are able to discriminate orientations against a horizontal target grating at orientation differences above 11° (Andermann et al., 2010), but no oblique target gratings were tested. In our touchscreen chamber, mice showed slightly higher discrimination thresholds between 15° and 25° at a mean of 23° for reliable discrimination. A potential reason for this small difference is that mice in the touchscreen chamber can freely move their head and body and may therefore not ideally observe both gratings in every single trial. This could be resolved through recent advancements in automated body tracking (Mathis et al., 2018), which allow to track the animals position and posture in real time and only present the visual stimuli when mice are in a specific location in front of the screen (Schneider et al., 2022). Head-fixed mice performed more trials (average 299 ± 57 trials) compared to our freely moving mice, which performed 125 ± 2.9 trials in the staircase experiment and 74 ± 2.5 trials in the parallel visual discrimination task.

### Increased response time with oblique discrimination and the role of visual attention

The animals’ response time was higher for oblique vs. cardinal stimulus sets in the middle period of training when performance differences were also the most prominent. Longer response times may thus reflect a higher discrimination difficulty with oblique orientations (Sanders and Kepecs, 2012; Ganea et al., 2020). However, this was not reflected in the response time during the staircase experiment where response times were largely similar for easy vs. harder orientation differences (Supplementary Figure 1B).

A possible explanation for this result is that we did not enforce a short response window in our task design. Since mice had 120 s to respond to the stimulus, there was little pressure to be highly attentive and sometimes mice were also resting in between trials. To leverage response times as a more accurate measure of visual attention and discrimination difficulty, it would therefore be advantageous to impose shorter response windows that force mice to be more attentive and quickly respond to solve the task. This could also be combined with above mentioned body-tracking approach to enforce a well-defined starting point that allows animals to quickly perceive and respond to the visual stimulus.

Given that visual attention is inherently harder to control in a freely moving vs. a head-fixed approach, our flexible task design could also be used to integrate additional spatial cues before or during the stimulus presentation. Providing such additional spatial cues would allow the implementation of Posner-style attention tasks in freely-moving mice that can serve to enforce and measure visual attention and its effect on sensory processing (You and Mysore, 2020; Li et al., 2021).

### Effects on discrimination performance due to retraining

To test if mice achieve higher perceptual accuracy with cardinal vs. oblique orientations, we first trained them with cardinal stimuli, similar to previous studies (Andermann et al., 2010). Mice were then retrained with oblique stimuli, resulting in larger orientation discrimination thresholds compared to cardinal orientations. However, a second cohort of mice that was directly trained with oblique stimuli achieved significantly lower orientation discrimination thresholds, resembling the orientation discrimination performance with cardinal gratings in the first group. These results demonstrate that the training procedure has a strong impact on the animals’ task performance and the obtained measures of visual perception. In addition, task learning had a strong inter-individual variability: while some mice learned the task within 10 sessions, others needed more than twice as long. Moreover, we found slight differences in learning speed and peak performance between male and female mice during the parallel learning discrimination learning, further emphasizing the need to account for factors that could cause inter-individual differences. Further investigations on the variability between littermates, sexes, and ages might therefore be beneficial for future operant conditioning studies to better understand and control for these effects. A higher level of standardization and automatization in the operation of behavioral platforms could also help to reduce variance and shed more light on questions about differences between individuals and groups of animals.

Previous studies that combined behavioral and two-photon imaging experiments in V1 showed a training-induced shift in the neural tuning towards the rewarded stimulus (Poort et al., 2015; Goltstein et al., 2018; Henschke et al., 2020) as also observed in electrophysiological studies (Schoups et al., 2001) The percentage of neurons responding to the rewarded stimulus orientation also increased after learning the task. Training the animal to respond to either cardinal or oblique targets, therefore, is likely to induce a similar change in the neuronal representations of these stimuli. Such learning-related effects could mask the innate distribution of neuronal tuning properties that are matched to the environment. To resolve this issue, we, therefore, employed a new experimental strategy where we trained mice on both targets simultaneously in a paired discrimination task. While mice eventually reached similar performance levels for both stimulus pairs, the learning duration was significantly longer for oblique compared to cardinal gratings. This suggests that training increased the sensitivity or the number of neurons that responded to oblique gratings to perform the task successfully. The innate oblique effect is therefore likely due to an adaptation of the visual cortex to the statistics of the environment but can be flexibly readjusted by experience, even in adult mice. Future studies will address this important question in more detail by imaging the tuning properties of V1 neurons before, during, and after parallel discrimination training.

### Flexible Python-based touchscreen chamber for operant conditioning

Our touchscreen chamber provides a comprehensive framework for operant conditioning of mice. The design provides a flexible behavioral setup, which can be easily adapted to different visual and/or auditory detection and discrimination tasks and combines the high demand for automatized, standardized, and flexible animal training. All hardware parts and software-solutions are freely available, thus enabling high reproducibility and collaboration across behavioral labs. Our setup is based on comparable designs that were used to study natural image perception in mice (Stirman et al., 2016; Yu et al., 2018). While the hardware design of the touchscreen chamber is comparable, we have extended the flexibility of our software solution to enable studies of complex sensory perception, e.g., with broadband visual stimuli, which consist of a distribution of orientations and spatial frequencies instead of standard gratings with a fixed spatial frequency and orientation (Simoncini et al., 2012). Our flexible software allows the seamless implementation of different experimental protocols, such as the staircase procedure for the fine-scaled quantification of perceptual thresholds, and the parallel visual discrimination task. This allows the parallel testing of stimulus perception in the same animals, therefore avoiding potential confounds from retraining or inter-individual variability. The flexibility in task design and creating complex sensory stimuli, is an important feature to answer future questions on sensory perception and behavior.

Our touchscreen chamber could also be used in labs that already operate commercial systems. While using commercial hardware directly with our open-source software would be highly limited by compatibility problems, the learning and task performance with our setup was similar to earlier studies that used commercial designs (Turner et al., 2017; Pak et al., 2020). Behavioral labs could, therefore, extend their infrastructure with new open-source touch screen chambers while still operating their existing commercial systems in parallel. The hardware could also be extended to use RFID-gated tunnels to integrate the chamber with automated homecage systems. This would allow mice to enter and leave the touchscreen chamber without human interference and enable more standardized studies with higher throughput.

The primary goal of our proposed system is to lower the hurdles for efficient behavioral experiments and support open science. To achieve an optimal level of certainty and usability, behavioral experiments should follow the following rules: standardized and automatized experiments, a non-aversive and low-stress environment, adaptivity to approaches in human psychophysics, the flexibility to use different paradigms, and the ability to record quantitative measurements (Mar et al., 2013). The chamber is mobile to share between collaborating partners and has also been used in undergraduate education courses to teach students how to use mice in psychophysics experiments achieving high data gain with low interference and stress-levels for the animals (Wiesbrock, 2020).

While head-fixation offers tight control of the sensory stimulus presentation and the motor response of the animal (Musall et al., 2019; Bjerre and Palmer, 2020) it is a more stressful procedure for the mouse compared to freely moving behaviors in a test chamber (Schwarz et al., 2010). Moreover, the loss of vestibulo-ocular feedback from head movements might strongly alter visual processing and explain why many freely-moving visual tasks are much harder to implement in head-fixed mice. Lastly, an increasing amount of neuronal recording techniques are optimized for freely-moving mice, such as chronically implanted high-density electrophysiology probes (Juavinett et al., 2019; Steinmetz et al., 2021) or head-mounted miniature calcium imaging devices (Aharoni et al., 2019).

## Conclusion

Studying sensory perception requires experimental settings that reflect the complexity of the environment, but naturalistic stimuli are inherently difficult to quantify or standardize across behavioral tasks. Using artificially designed visual stimuli that reflect the statistics of the natural environment allows us to test the visual system under the conditions it is optimized for (Simoncelli and Olshausen, 2001; Balla et al., 2021). Here, we demonstrate that mice, like humans, show improved perception of cardinal edges. This can be explained by the larger percentage of neurons responding to this visual feature which is an adaptation of the visual system to the natural visual environment (Girshick et al., 2011; Kreile et al., 2011; Roth et al., 2012). Aside from using complex visual stimuli, it is important to consider the behavioral setting when testing perceptual performance. Training experience also has a strong impact on their sensory perception (Schoups et al., 2001; Poort et al., 2015; Goltstein et al., 2018; Henschke et al., 2020). Therefore, the behavior task and training strategy should compare the perception of different stimuli under the same conditions. Mice also express large inter-individual behavioral variance even if they are largely genetically identical (Freund et al., 2013). Within-subject comparisons of different target stimuli are, therefore, required as shown in our paired visual discrimination task. Provided completely open-source, our flexible touchscreen chamber allows non-invasive testing of natural behavior and perception in mice with high flexibility and reproducibility across labs further supporting community sharing of behavior data obtained in touchscreen chambers (Beraldo et al., 2019).

## Data Availability Statement

The raw data supporting the conclusions of this article will be made available by the authors, without undue reservation.

## Ethics Statement

The animal study was reviewed and approved by Landesamt für Natur, Umwelt und Verbraucherschutz Nordrhein-Westfalen.

## Author Contributions

CW and BK contributed to the conception and design of the study. CW performed the experiments and the statistical analysis and wrote the first draft of the manuscript. CW, SM, and BK wrote sections of the manuscript. All authors contributed to the article and approved the submitted version.

## Funding

This work was funded by the Deutsche Forschungsgemeinschaft (DFG, German Research Foundation) - 368482240/GRK2416.

## Footnotes

^1^ https://github.com/BRAINLab-Aachen/Touchscreen-Chamber

## Supplementary Material

Supplementary Figure 1 **(A)** Distribution of trials per orientation difference. The different trial numbers are explained by the different number of sessions performed in each learning condition. **(B)** Linear fit between response delay and orientation difference. A significant negative correlation is shown for the cardinal learning condition (Wald Test, *p* = 0.024, retraining: *p* = 0.31, oblique: *p* = 0.62).
