# Supplementary figures and images for: A flexible Python-based touchscreen chamber for operant conditioning reveals improved visual perception of cardinal orientations in mice
Source: Front Cell Neurosci. 2022 Oct 10;16:866109. doi: 10.3389/fncel.2022.866109 (PMC9588922; doi:10.3389/fncel.2022.866109)

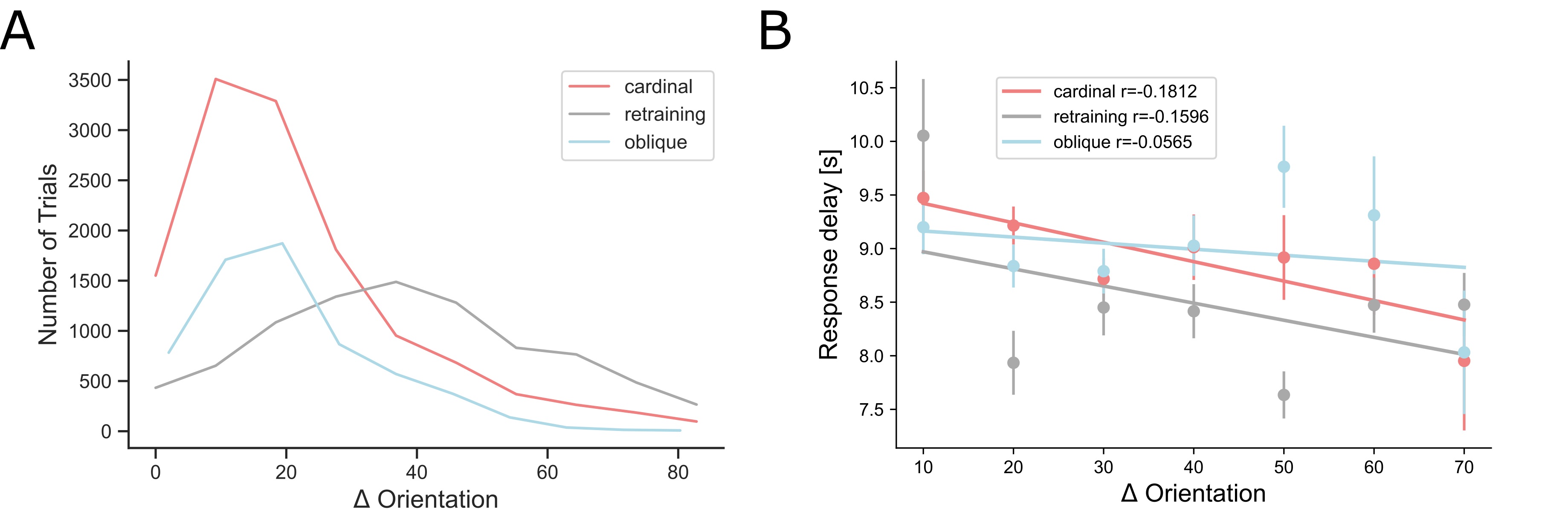

Supplement: Supplementary Figure 1 — (A) Distribution of trials per orientation difference. The different trial numbers are explained by the different number of sessions performed in each learning condition. (B) Linear fit between response delay and orientation difference. A significant negative correlation is shown for the cardinal learning condition (Wald Test, p = 0.024, retraining: p = 0.31, oblique: p = 0.62). [file Image_1.JPEG]
